# Grazing decreases net ecosystem carbon exchange by decreasing shrub and semi‐shrub biomass in a desert steppe

**DOI:** 10.1002/ece3.11528

**Published:** 2024-06-25

**Authors:** Xin Ju, Bingying Wang, Lianhai Wu, Xiaojia Zhang, Qian Wu, Guodong Han

**Affiliations:** ^1^ Key Laboratory of Grassland Resources of the Ministry of Education, Key Laboratory of Forage Cultivation, Processing and High Efficient Utilization of the Ministry of Agriculture and Rural Affairs, Inner Mongolia Key Laboratory of Grassland Management and Utilization, College of Grassland, Resources and Environment Inner Mongolia Agricultural University Hohhot Inner Mongolia China; ^2^ Forest and Grassland Protection and Development Center Bairin Right Banner Inner Mongolia China; ^3^ Net Zero and Resilient Farming Rothamsted Research Devon UK

**Keywords:** ecosystem respiration, grazing intensity, net ecosystem carbon exchange, soil respiration, *Stipa breviflora* desert steppe

## Abstract

Livestock grazing can strongly determine how grasslands function and their role in the carbon cycle. However, how ecosystem carbon exchange responds to grazing and the underlying mechanisms remain unclear. We measured ecosystem carbon fluxes to explore the changes in carbon exchange and their driving mechanisms under different grazing intensities (CK, control; HG, heavy grazing; LG, light grazing; MG, moderate grazing) based on a 16‐year long‐term grazing experimental platform in a desert steppe. We found that grazing intensity influenced aboveground biomass during the peak growing season, primarily by decreasing shrubs and semi‐shrubs and perennial forbs. Furthermore, grazing decreased net ecosystem carbon exchange by decreasing aboveground biomass, especially the functional group of shrubs and semi‐shrubs. At the same time, we found that belowground biomass and soil ammonium nitrogen were the driving factors of soil respiration in grazed systems. Our study indicates that shrubs and semi‐shrubs are important factors in regulating ecosystem carbon exchange under grazing disturbance in the desert steppe, whereas belowground biomass and soil available nitrogen are important factors regulating soil respiration under grazing disturbance in the desert steppe; this results provide deeper insights for understanding how grazing moderates the relationships between soil nutrients, plant biomass, and ecosystem CO_2_ exchange, which provide a theoretical basis for further grazing management.

## Graphical abstract

We measured ecosystem carbon fluxes and their associations in response to a long‐term (16‐year) grazer manipulation experiment in a desert steppe grassland in Inner Mongolia, China. Our results show that grazers alter patterns of net ecosystem exchange primarily via their negative influence on the biomass of shrubs and subshrubs, which play a prominent role in ecosystem functions.

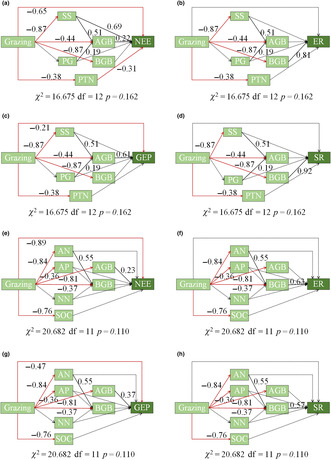

## 1 INTRODUCTION

Grassland ecosystems cover a large proportion of the arid and semi‐arid regions of the world, playing an important role in the global carbon cycle (Lei et al., 2020; Schuman et al., 2002; Scurlock & Hall, 1998; Zhou et al., 2019). The degree to which terrestrial ecosystems serve as net carbon sinks or sources depends on the balance between the carbon fixed by plant photosynthesis and the carbon released into the atmosphere by plant and soil respiration (Jin et al., 2023; Li, Han, et al., 2017; Peng et al., 2014). While the degree to which forested systems serve as net carbon sinks or sources has been well studied (Martens et al., 2004; Rebane et al., 2020), the role of grassland ecosystems as carbon sources or sinks can be highly variable (Chang et al., 2021; Dai et al., 2014; Smith, 2014). Grasslands can serve as an important carbon sink (Hafner et al., 2012; Sha et al., 2020), a net carbon source (Kuzyakov & Gavrichkova, 2010), neither a source nor sink, which be in equilibrium (Hao et al., 2017), or can fluctuate between states (Dai et al., 2014; Zhang et al., 2020). As a result, the patterns of carbon exchange in grasslands remain an area in need of exploration.

Livestock grazing is a significant land‐use category by which human activities can influence the structure and function of grassland ecosystems, profoundly altering the carbon cycle and stability of grassland productivity (Zhang, Bennett, et al., 2023; Zhang, Zheng, et al., 2023). Grazing directly affects plant productivity because livestock remove leaves and stems, promote compensatory growth, redistribute soil organic matter, and alter soil respiration via their trampling and excrement (Barthelemy et al., 2018; Cao et al., 2004; Chen et al., 2015; Veldhuis et al., 2018). Grazing also alters soil nitrogen content and other processes important to the carbon cycle, such as litter decomposition and photosynthate distribution (Xia & Wan, 2008). As a result, grazing can moderate the net ecosystem exchange of grasslands and whether they serve as a net carbon sink or source. In some cases, light to moderate levels of grazing can facilitate grasslands being net carbon sinks (Chang et al., 2021; Derner et al., 2006; Sha et al., 2020), while high levels of grazing can accelerate the release of carbon and switch the ecosystem to a carbon source (Liang et al., 2017; Tang et al., 2018). In other cases, grazing can have little influence on the carbon budget of grassland ecosystems (Fang et al., 2010; Piñeiro et al., 2010). To explore how grazing influences the patterns and mechanisms of carbon exchange in grassland ecosystems, it is necessary to simultaneously consider the impact of grazing livestock on both vegetation and soil.

Desert steppe is particularly vulnerable to degradation due to livestock grazing, which along with other disturbances, can transition them from carbon sinks to carbon sources (Zhang et al., 2020). In our study area, we assessed ecosystem carbon balances over a sustained 10‐year period and explored the influencing factors; we concluded that both precipitation patterns and grazing can combine to cause changes to the carbon sink in a desert steppe (Jin et al., 2023; Wang et al., 2023), but ecosystem carbon exchange is influenced by a combination of environmental (soil, climate) and biological (grazing) factors. How these l factors influence net ecosystem carbon exchange depends on the relationship between carbon uptake via primary productivity and carbon release via plant and soil respiration. Furthermore, there is considerable uncertainty regarding the factors influencing carbon exchange in grassland ecosystems (Liu, van Dijk, et al., 2015; Sha et al., 2020). This is likely because the variability in ecosystem carbon exchange is mediated by grassland types, climate, vegetation, and soil (Helfter et al., 2015; Hussain et al., 2015; Liang et al., 2020), as well as by grazing practices (Dai et al., 2014; Fang et al., 2010).

Thus, simply measuring net ecosystem exchange and aboveground biomass is not enough to fully understand the influence of biotic and abiotic factors on these rates (Bajgain et al., 2018; Li, Wu, et al., 2017). By identifying how carbon exchange and soil respiration are influenced by grazing and environmental factors, we can better understand the factors influencing carbon dynamics in these important ecosystems, and use this information to develop policies for the sustainable management and conservation of grassland resources. In this study, we measured ecosystem carbon fluxes and their associations in response to a long‐term (16‐year) grazer manipulation experiment in a desert steppe grassland in Inner Mongolia, China. We specifically asked (1) how grazing influences features of the plant community and soil conditions and (2) how those effects influence the parameters of net ecosystem carbon exchange, including gross ecosystem productivity and respiration. Based on our previous research, we further measured above‐ and belowground biomass, plant nutrients (carbon and nitrogen content of plant communities), and soil nutrient indices to analyze the main drivers influencing the exchanges of CO_2_ fluxes in desert steppe and their responses to grazing disturbances. Overall, our current study aims to fill this gap of whether environmental factors, grazing livestock disturbance of grassland vegetation and soils combine to regulate ecosystem carbon exchange by utilizing a long‐term field experiment of different grazing intensities and to provide a theoretical basis for the adaptive management of desert steppe.

## 2 METHODS

### 2.1 Study site

Our study took place within a long‐term grazing experiment located in Siziwang Banner (41°46′43″ N, 111°53′42″ E, elevation 1456 m) at the comprehensive experiment and demonstration center of the Inner Mongolia Academy of Agriculture and Animal Husbandry Sciences, China. The study site is a typical desert steppe ecosystem dominated by *Stipa breviflora* Griseb., *Artemisia frigida* Willd, and *Cleistogenes songorica* (Roshev.) Ohwi. Subordinate species include *Convolvulus ammannii* Desr., *Kochia prostrata* (L.) Schrad., *Caragana stenophylla* Pojark., and *Caragana microphylla* Lam. The soil is primarily a sandy loam texture with low nitrogen, phosphorus, and organic matter content, but high potassium. Over the course of the experiment (2004–2020), the average annual temperature was 3.4°C, and the average annual precipitation was 221.7 mm (the majority falling from June to August). We present the air temperature and precipitation during the growing season in which we collected data (2020) in Figure S1.

### 2.2 Experimental design

Prior to 2004, this study site was grazed year‐round by sheep at a relatively high stocking rate (~1.0 sheep equivalent ha^−1^) (Kemp et al., 2013), leading to a relatively degraded grassland with 17%–20% vegetative cover (Wang, Jiao, et al., 2014). A grazing manipulation experiment was established in June 2004 in a ~50 ha site with relatively flat terrain and homogeneous vegetation and soil types. Twelve experimental plots of 4.4 ha were constructed with iron wire fencing and distributed among three replicate experimental blocks, which each received one of four grazing treatments: control (no grazing), light grazing (0.91 sheep unit · [hm^2^ A^−1^] ^−1^), moderate grazing (1.82 sheep unit · [hm^2^ A^−1^] ^−1^), and heavy grazing (2.71 sheep unit · [hm^2^ A^−1^] ^−1^). These grazing intensities were referred to the theoretical stock capacity of *Stipa breviflora* desert steppe and the design proposed by Wei et al. (2000). Each grazed plot was grazed by adult sheep from June 1 to October 1 each year. During the grazing season, the sheep were driven into the plot at 6:00 every day and left to forage freely until their return to the corral at 18:00.

### 2.3 Measurement of aboveground biomass and belowground biomass

We measured aboveground biomass of plants monthly from June to September 2020. In each month, we randomly selected three 1 m^2^ quadrats (108 quadrats in total) near the other sampling locations in each plot to record the community characteristics of plants. In each quadrat, we clipped all aboveground plants and separated them to species. We then dried plants at 65°C for 48 h and weighed them. We categorized species into four functional groups (Bai et al., 2010), including (1) perennial grass, (2) shrub and semi‐shrub, (3) perennial forb, and (4) annual and biennial plants (Table S1).

In August 2020, we measured belowground biomass. To do so, we selected six points near the other sampling locations and collected samples from the 0–10 cm layer with a root auger (7 cm diameter). We took two samples at each point and combined them for analysis. We picked roots from the soil, washed them, and dried and weighed them as above.

### 2.4 Measurement of plant total nitrogen and carbon content

We measured total carbon and total nitrogen contents from three of the aboveground sampling quadrats in each plot. To do so, after weighing, we mixed all the aboveground plants cut in the quadratand, and subsequently, we ground tissues using a ball mill and measured powder samples using an elemental analyzer (Elementar Vario MACRO CUBE).

### 2.5 Measurement of soil properties

We determined several soil physical and chemical properties in August 2020 from soil samples. We selected six points in each plot near the other sampling points and collected soil at each point from the 0–10 cm layer using a soil auger (3 cm diameter). At each point, we collected two soil samples, combined them, and passed them through a 2 mm sieve to determine the physical and chemical properties of the soil in the laboratory.

For each soil sample, we determined the total carbon and total nitrogen content in the soil using an elemental analyzer (Elementar Vario MACRO CUBE); total phosphorus content using an ultraviolet spectrophotometer (UV‐1800, Mapada, Shanghai, China) with the sodium hydroxide fusion method; organic carbon content using the potassium dichromate external heating method; nitrate (${\mathrm{NO}}_{3}^{-}$‐N) and ammonium (${\mathrm{NH}}_{4}^{+}$‐N) by extraction using KCl (2 mol·L^−1^) with a flow analyzer; available phosphorus content using the sodium bicarbonate molybdenum antimony anti‐colorimetric method; and microbial biomass carbon and microbial biomass nitrogen using the chloroform fumigation extraction method.

### 2.6 Measurement of ecosystem CO_2_ exchange

We measured net ecosystem CO_2_ exchange and ecosystem respiration monthly during the growing season (June to October) in 2020. To do so, we used a Li‐6400 portable photosynthetic (Li‐COR, USA) instrument with the static chamber method. We collected measurements between 8:00 a.m. and 12:00 p.m. on a clear cloudless and windless day (as much as possible), at least 3 days after a rainfall (Niu et al., 2008; Wu et al., 2021). For measurements, we connected a leaf chamber (50 × 50 × 50 cm^3^ transparent plexiglass box) to the portable photosynthetic instrument and installed a small fan in each diagonal direction at the upper end of the glass box to mix the gas. We placed the glass box on one of three aluminum sink frames (50 × 50 cm^2^) placed randomly within each plot to ensure an airtight seal. We repeated measurements on each of the three frames.

At each sample point, the measurement time was 120 s, and CO_2_ concentration and water exchange flux values were automatically recorded every 10 s. After these measurements, we ventilated the leaf chamber to ensure it was filled with convection‐exchanged air, covered it with a black cloth to ensure no light transmission, and repeated the above procedure to determine ecosystem respiration.

We measured soil respiration (SR) using an open circuit Li‐8100 soil carbon flux meter (Li‐COR, USA) at the same time as the net ecosystem exchange measurements. We measured soil respiration within three PVC rings (10.5 cm in diameter and 8 cm in height) that were randomly placed 2 cm above the ground surface in each plot. Prior to measurements, we clipped plants inside the rings flush with the ground and removed debris.

We calculated net ecosystem CO_2_ exchange (NEE) and gross ecosystem productivity (GEP), given ecosystem respiration (ER), as follows: ∂*C*′ ∂*t* = INDEX(LINEST(*Y*1: *Y*12, *A*1: *A*12),1); $\mathrm{NEE}=\frac{10\mathrm{VP}\left(1-\frac{W}{1000}\right)}{\mathrm{RS}\left(T+273.15\right)}\frac{\partial C^{\prime }}{\partial t}$; NEE = GEP − ER.

Units for NEE, ER, and GEP are μmol·m^−2^·s^−1^. *Y*1‐*Y*12 is the CO_2_ concentration value, *A*1‐*A*12 is the measurement time, *V* represents the volume of the box (cm^3^), *P* is the atmospheric pressure inside the box (kpa), *W* is the water pressure inside the chamber (mmol·mol^−1^), *S* is the bottom area of the chamber (cm^2^), *T* is the temperature of the gas inside the chamber (°C), and *R* = 8.314 J·mol^−1^·K^−1^ (constant). We used values of ecosystem CO_2_ exchange and soil respiration during the growing season (June–October) to calculate the values of NEE, GEP, ER, and SR for each treatment.

### 2.7 Measurement of air temperature and precipitation

We collected meteorological data in 2020 using a small weather station (Gro Weather software version 1.2, Davis Instruments Corporation, USA). The station automatically recorded temperature and precipitation data at 1 h intervals, which we downloaded and collated at regular intervals.

### 2.8 Measurement of soil temperature and moisture

In parallel with net ecosystem exchange measurements, we measured soil temperature at 10 cm depth in the leaf chamber with two TP3001 electronic thermometers. At the same time, we collected 10 cm soil samples using a 2.5 cm diameter × 10 cm high soil auger, which we collected in an aluminum box, weighed and recorded the wet mass, and then dried at 105°C for 24 h to weigh the dry mass and then calculate the mass water content.

### 2.9 Data analysis

After ensuring data met normality and homogeneity of variance assumptions using the Shapiro–Wilk test, we evaluated the influence of grazing treatment on above‐ and belowground biomass, plant nitrogen and carbon content, plant functional groups, and several soil chemical variables, and the ecosystem CO_2_ exchange and soil respiration. To do so, we used repeated measures ANOVA to test the effects of grazing intensity and sampling month on the aboveground biomass, plant functional group biomass, ecosystem CO_2_ exchange and soil respiration. We used one‐way ANOVA followed by a Duncan test for pairwise comparison to test the effects of grazing intensity on the belowground biomass, plant total carbon, plant total nitrogen, and soil nutrient content. A *p* < .05 indicated significance in the treatment effects.

We correlated several abiotic factors with ecosystem carbon exchange, including temperature, precipitation, soil temperature, and soil moisture in each treatment using regression analysis.

To investigate the influence of soil and plant factors on ecosystem carbon exchange, we used redundancy analysis to rank the impact of the factors on carbon exchange. Furthermore, we performed Pearson's correlation analyses. Based on the results of RDA analysis and correlation analysis, we used a generalized linear model (GLM) and structural equation model (SEM) to determine the effects of plant and soil factors on ecosystem CO_2_ exchange and soil respiration. To do so, we first calculated the contribution of the plant and soil factors on the ecosystem CO_2_ exchange and soil respiration using the GLM and correlation analyses, and then we removed insignificant pathways and simplified the SEM model based on the GLM and correlation analysis results. We obtained path coefficients using a maximum likelihood estimation technique.

We performed ANOVA, repeated measures ANOVA and the GLM analyses in version R 4.0.3. The SEM analyses were performed using the “piecewise SEM” package (Lefcheck, 2016) in R version 4.0.3. We performed regression analyses, redundancy analyses, and Pearson's correlation analyses and plots using Origin 2023 software.

## 3 RESULTS

### 3.1 Grazing intensity effects on the plant functional group productivity and plant community carbon and nitrogen content

**FIGURE 1:**
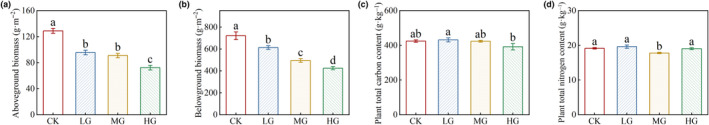
The effects of grazing intensity on plant aboveground biomass (a), belowground biomass (b), plant community carbon content (c), and plant community nitrogen content (d). Different lowercase letters indicate significant differences between means at *p* < .05. Error bars are ±SE. Codes of different treatments are as follows: CK, control/no grazing; HG, heavy grazing; LG, light grazing; MG, moderate grazing.

We found that both aboveground (Figure 1a) and belowground (Figure 1b) biomass was influenced by the grazing treatment. All grazing treatments had lower aboveground and belowground biomass than the control treatment with no grazing. Aboveground biomass was lowest in the HG treatment, while there were no differences between LG intensity and MG treatments (*p* > .05, Figure 1a). Belowground biomass was incrementally lower as grazing intensity increased (*p* < .05, Figure 1b). When we analyzed differences in nutrient content, we found that the total carbon content of the plant community was lowest in the HG treatment (Figure 1c), while the total nitrogen content of the plant community was lowest in the MG treatment (Figure 1d). Aboveground biomass differed significantly between months (*p* < .01), though the difference was not significant for the interaction between month and grazing intensity (*p* > .05, Table 1).

**TABLE 1:** Repeated‐measures ANOVA for aboveground biomass and biomass of plant functional groups.

Plant biomass	Month			Grazing intensity			Month × grazing intensity		
	*F* value	*p* value	df	*F* value	*p* value	df	*F* value	*p* value	df
AGB (g·m^−2^)	6.59	.002	3	10.91	.003	3	0.63	.75	9
PG (g·m^−2^)	1.78	.18	3	1.77	.24	3	0.53	.83	9
SS (g·m^−2^)	4.22	.02	3	10.62	.004	3	2.22	.05	9
PF (g·m^−2^)	9.74	<.001	3	8.28	.008	3	3.96	.003	9
AB (g·m^−2^)	19.62	<.001	3	4.66	.025	3	0.97	.49	9

*Note*: The *F* values are presented together with their levels of significance and degree of freedom. AGB, PG, SS, PF, and AB represent aboveground biomass, perennial grass biomass, shrub and semi‐shrub biomass, perennial forb biomass, and annual and biennial plant biomass.

**FIGURE 2:**
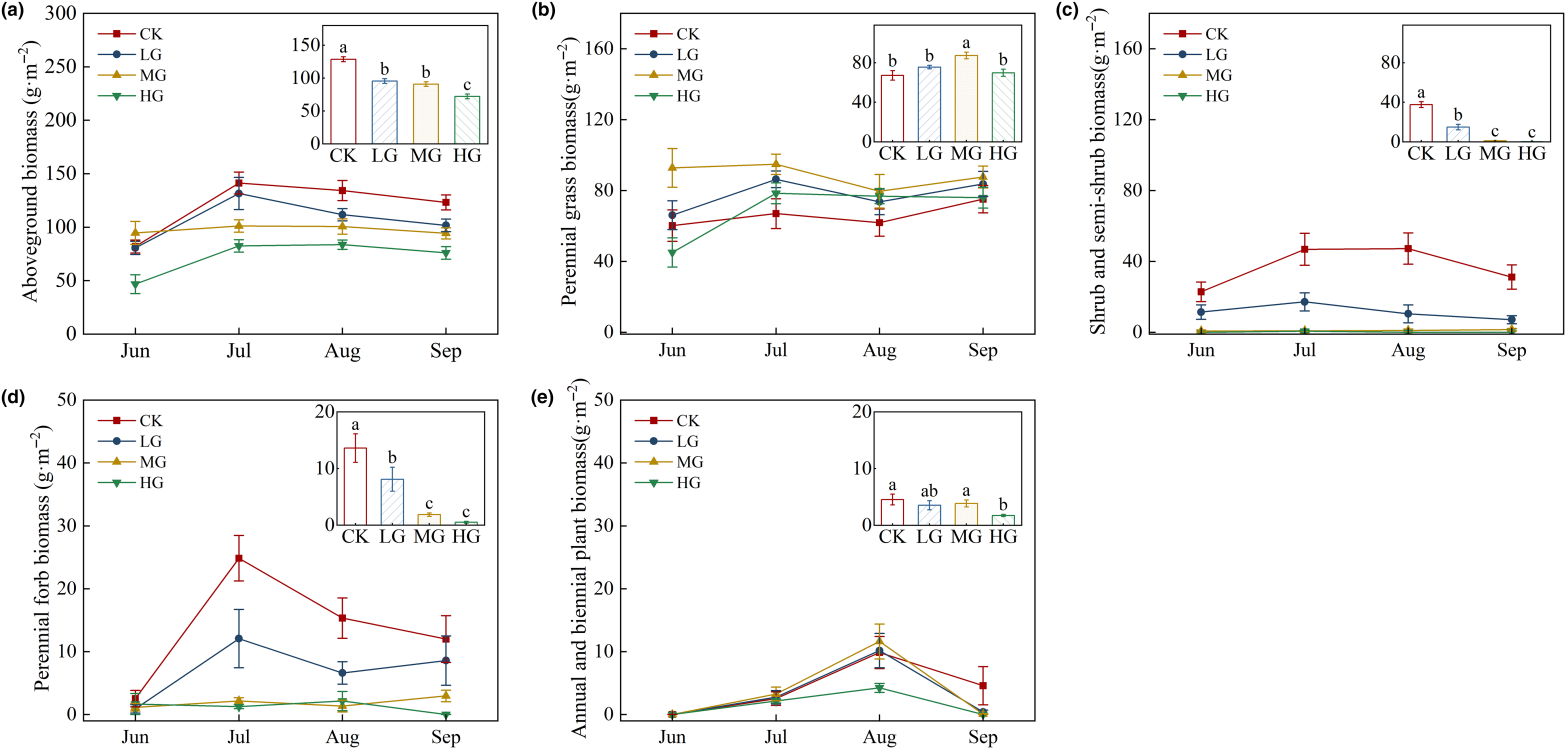
The effects of grazing intensity and month on plant above ground biomass (a), perennial grass biomass (b), shrub and semi‐shrub biomass (c), perennial forb biomass (d), annual and biennial plant biomass (e). Each panel represents a different grouping of plant biomass. Different lowercase letters indicate significant differences between means at *p* < .05. Error bars are ±SE, and the lines in panels (b–e) show the biomass of each plant functional group during the 2020 growing season. Codes of different treatments are as follows: CK, control/no grazing; HG, heavy grazing; LG, light grazing; MG, moderate grazing.

When we divided plants into functional groups (Figure 2b–e), we found that most groups strongly declined with increasing grazing, particularly shrubs and semi‐shrubs (*p* < .05, Figure 2c), as well as perennial forbs (*p* < .05, Figure 2d). Perennial grasses had greater biomass in the MG treatment (*p* < .05, Figure 2b). Using repeated measures ANOVAs for different plant functional groups, we found that perennial forbs, as well as shrubs and semi‐shrubs, differed significantly by month, grazing intensity, and the interaction between month and grazing intensity (*p* < .05). There was no interaction between month and grazing intensity for annuals and biennials (*p* > .05), while there were no main or interactive effects on month or grazing intensity on perennial grasses (*p* > .05, Table 1, Figure 2b–e).

### 3.2 Grazing intensity effects on soil nutrients

**FIGURE 3:**
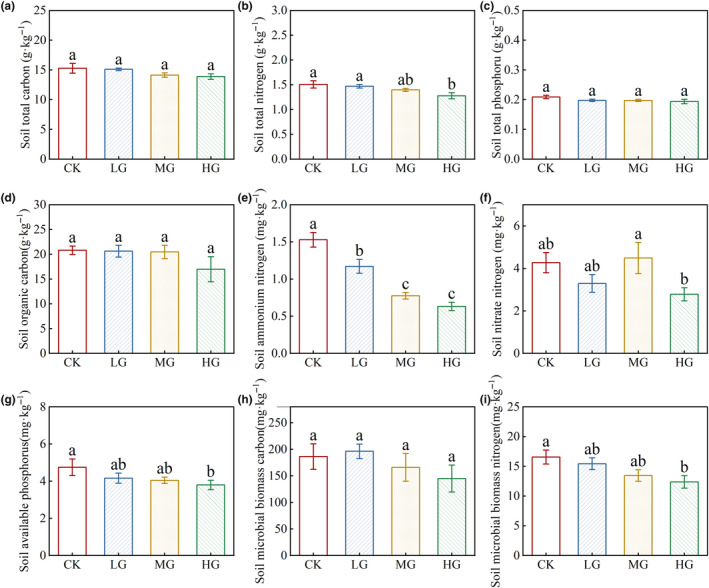
The effects of grazing intensity on soil total carbon (a), soil total nitrogen (b), soil total phosphorus (c), soil organic carbon (d), soil ammonium nitrogen (e), soil nitrate nitrogen (f), soil available phosphorus (g), soil microbial biomass carbon (h) and soil microbial biomass nitrogen (i). Different lowercase letters indicate significant differences between means at *p* < .05. Error bars are ±SE. Codes of different treatments are as follows: CK, control/no grazing; HG, heavy grazing; LG, light grazing; MG, moderate grazing.

Of the soil chemical variables, we found no differences in total carbon (Figure 3a), total phosphorus (Figure 3c), organic carbon (Figure 3d), and microbial biomass carbon (Figure 3h) among grazing intensity treatments (*p* > .05). However, we found significantly lower levels of total nitrogen (Figure 3b), ammonium nitrogen (Figure 3e), microbial biomass nitrogen (Figure 3i), and available phosphorus (Figure 3g) in the HG intensity treatments compared to no grazing (*p* < .05).

### 3.3 Differences in ecosystem CO_2_ exchange under different grazing intensities

**FIGURE 4:**
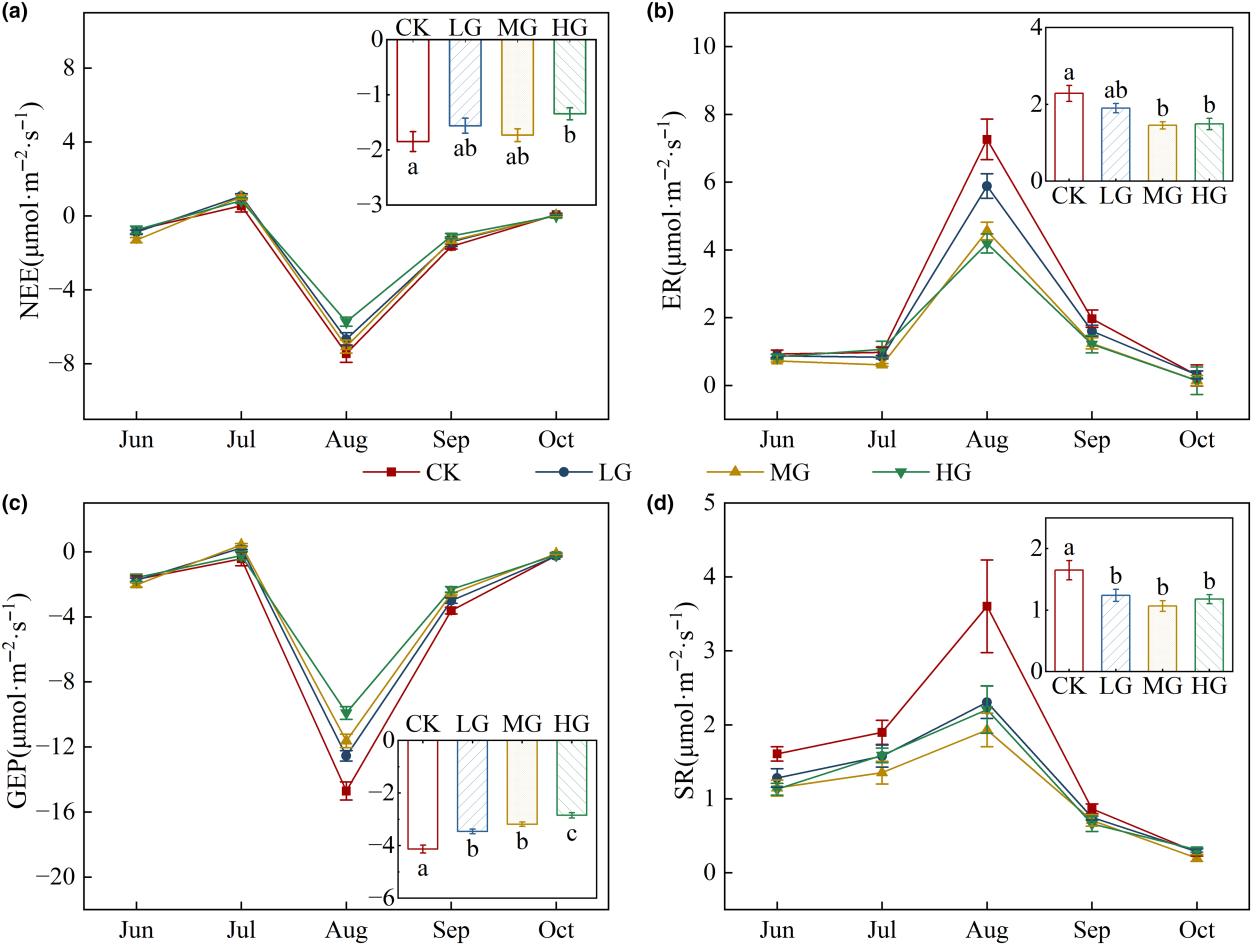
Monthly dynamics of ecosystem fluxes. Panels show the mean value (±SE) of net exchange of ecosystem CO_2_ (a, NEE), ecosystem respiration (b, ER), gross ecosystem productivity (c, GEP), and soil respiration (d, SR) in the growing season (June–October) of 2020. The inset reflects the differences between treatments in the 2020 growing season, where positive and negative values represent net carbon release and uptake by the ecosystem and do not indicate the magnitude of the values. Different lowercase letters indicate significant differences between treatments (*p* < .05). Codes of different treatments are the same as in Figure 3.

During the 2020 growing season, we found that NEE, ER, GEP, and SR showed significant seasonal patterns (Figure 4), as did precipitation and soil moisture (Figures S1 and S2). Variation in precipitation had a significant effect on NEE, GEP, and ER (*p* < .05), while variation in soil moisture had a significant effect on GEP (*p* = .002), ER (*p* < .001), and SR (*p* < .001, Figure S3). We found significant differences in NEE, ER, GEP, and SR across months (*p* < .001), while NEE and GEP also varied significantly between grazing intensities and the interaction between month and grazing intensity (*p* < .01); there were no effects of grazing intensity or the interaction with month for ER and SR (*p* > .05, Table 2). During July, NEE was positive, indicating carbon release as a source (Figure 4a). During the growing season, NEE was negative, indicating a carbon sink. NEE (Figure 4a), GEP (Figure 4c), ER (Figure 4b), and SR (Figure 4d) were highest in August. When we compared grazing treatments, we found that the rates of NEE (Figure 4a), ER (Figure 4b), GEP (Figure 4c), and SR (Figure 4d) were all significantly lower in the HG treatment compared to the no‐grazing treatment (*p* < .05).

**TABLE 2:** Repeated‐measures ANOVA for ecosystem carbon fluxes and soil respiration.

Ecosystem fluxes	Month			Grazing intensity			Month × grazing intensity		
	*F* value	*p* value	df	*F* value	*p* value	df	*F* value	*p* value	df
NEE (μmol·m^−2^·s^−1^)	1039.00	<.001	4	32.56	.004	3	7.59	<.001	12
ER (μmol·m^−2^·s^−1^)	190.52	<.001	4	2.28	.16	3	3.64	.06	12
GEP (μmol·m^−2^·s^−1^)	1082.33	<.001	4	40.77	<.001	3	8.02	<.001	12
SR (μmol·m^−2^·s^−1^)	48.76	<.001	4	1.98	.2	3	1.42	.21	12

*Note*: The *F* values are presented together with their levels of significance and degree of freedom. NEE, ER, GEP, and SR represent net exchange of ecosystem CO_2_, ecosystem respiration, gross ecosystem productivity, and soil respiration.

### 3.4 Effects of plant and soil factors on ecosystem CO_2_ exchange

**FIGURE 5:**
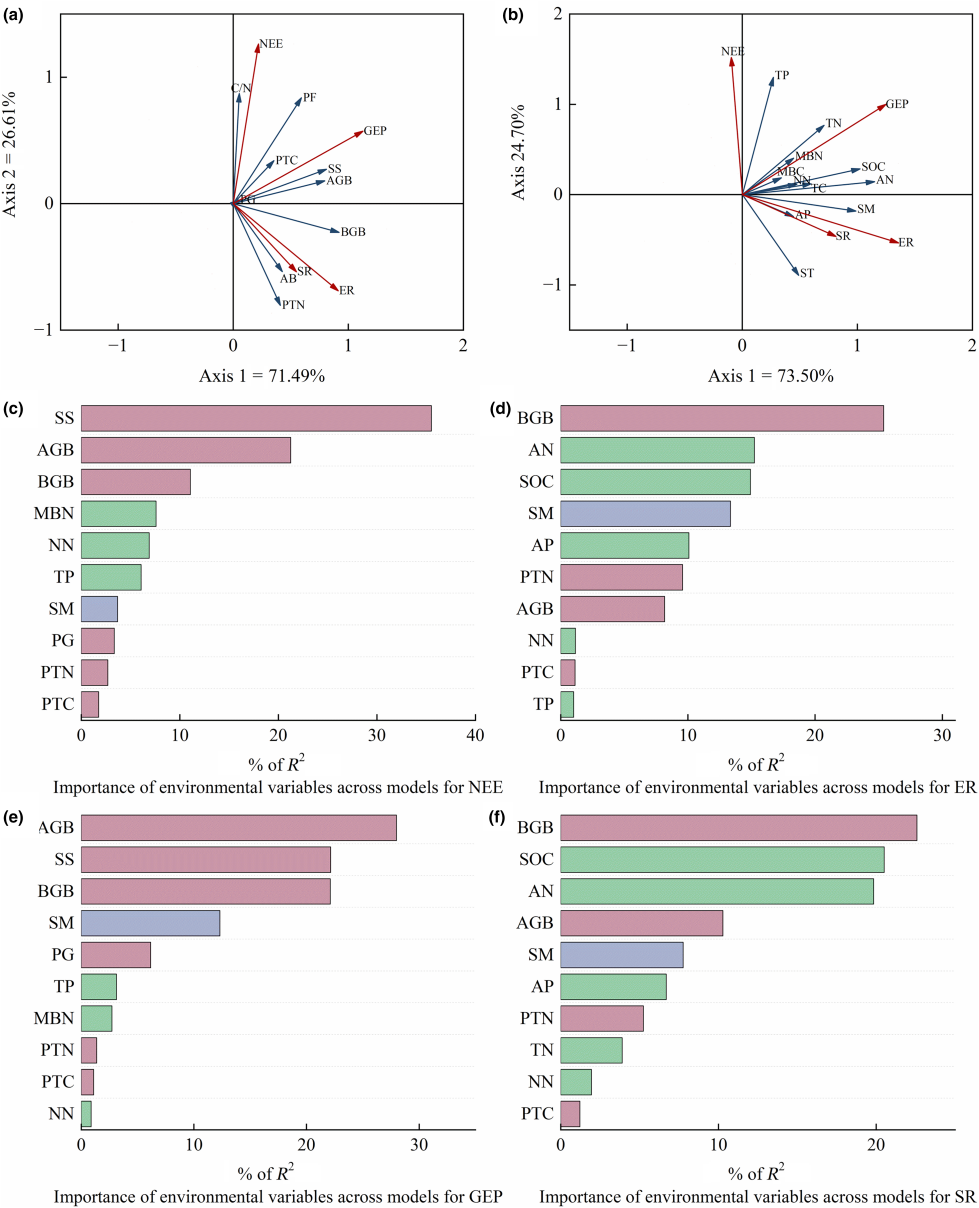
Biplot of ecosystem carbon exchange (NEE, ER, GEP, SR) from redundancy analysis (RDA) for plant factors (a) and soil factors (b). Generalized linear model (GLM) analysis was used to study the contribution of the plant and soil factors to the net exchange of ecosystem CO_2_ (c, NEE), ecosystem respiration (d, ER), gross ecosystem productivity (e, GEP), and soil respiration (f, SR). (a, b) Ecosystem carbon exchange is represented as red lines with arrows; plant factors (a) and soil factors (b) are represented as blue lines with arrows. The length of the line indicates the magnitude of the correlation between the explanatory variable and ecosystem carbon exchange. The angle between the lines indicates the correlation between the variables, and the angle between the red and blue arrows is less than 90° for positive correlations. Codes of different plant factors are as follows: AB, annual and biennial plant biomass; AGB, aboveground biomass; BGB, belowground biomass; C/N, the ratio of total plant carbon content to total plant nitrogen content; PF, perennial forb biomass; PG, perennial grass biomass; PTC, plant total carbon; PTN, plant total nitrogen; SS, shrub and semi‐shrub biomass. Codes of different soil factors are as follows: AN, ammonium nitrogen; AP, available phosphorus; MBC, microbial biomass carbon; MBN, microbial biomass nitrogen; NN, nitrate nitrogen; SOC, organic carbon; TC, total carbon; TN, total nitrogen; TP, total phosphorus. (c–f) The importance of individual environmental variables across models for ecosystem carbon exchange is shown for each indicator as variable importance weighted by % of *R* ^2^.

We used RDA to examine the relationship between the explanatory variables (plant and soil factors, blue lines with arrows) and response variables (ecosystem carbon exchange and soil respiration, red lines with arrows) in Figure 5. We found that plant factors (e.g., above‐ and belowground biomass, plant carbon, and nitrogen nutrients) explained 98.10% of the variance of ecosystem CO_2_ exchange and soil respiration (Axis 1 explained 71.49% of the total variance, whereas Axis 2 explained 26.61%; Figure 5a). Soil factors (e.g., soil nutrient index) explained 98.20% of the variance of ecosystem CO_2_ exchange and soil respiration (Axis 1 explained 73.50% of the total variance, whereas Axis 2 explained 24.70%; Figure 5b). For plant and soil factors, shrub and semi‐shrub biomass (*R* ^2^ = .36) contributed the most to variance of NEE, followed by aboveground biomass (*R* ^2^ = .21, Figure 5c); aboveground biomass (*R* ^2^ = .28) contributed the most to the variance of GEP, followed by shrub and semi‐shrub biomass (*R* ^2^ = .22, Figure 5e); belowground biomass (*R* ^2^ = .25, *R* ^2^ = .23) contributed the most to the variance of ER and SR (Figure 5d,f). According to Pearson's correlation analysis (Figure S4), we found that both aboveground biomass and shrub and semi‐shrub biomass showed significant positive correlations with NEE and GEP (*p* < .001), and belowground biomass, organic carbon, and ammonium nitrogen showed significant positive correlations with ER and SR (*p* < .001).

**FIGURE 6:**
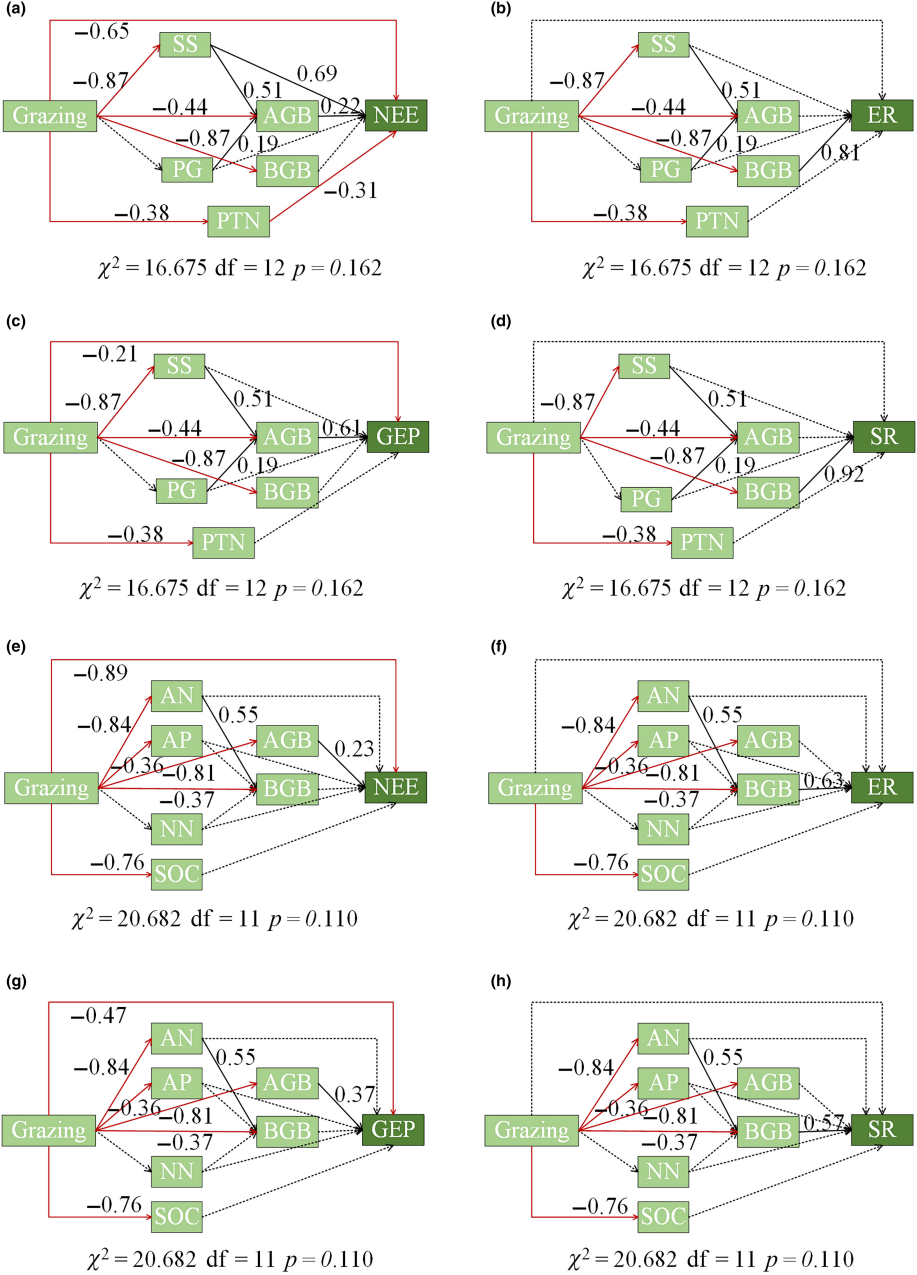
Structural equation models (SEMs) examining the standard total effects of plant factors on NEE (a), ER (b), GEP (c), SR (d), and soil factors on NEE (e), ER (f), GEP (g), SR (h) under different grazing intensities. Boxes stand for measured variables in the model. Standardized path coefficients are given. Solid black lines represent positive paths (*p* < .05), solid red lines represent negative paths (*p* < .05), and dotted black arrows represent nonsignificant paths (*p* > .05).

Based on the results of the redundancy and GLM analyses, we developed SEMs to better explain the driving mechanisms of ecosystem carbon exchange and soil respiration. Our SEM analysis showed that grazing had a direct negative effect on NEE and GEP. Specifically, grazing reduced NEE and GEP by reducing aboveground biomass, particularly through the indirect reduction of NEE due to lower shrub and semi‐shrub biomass (Figure 6a,c). However, the lower soil nutrient content in the grazing treatment was not associated with NEE and GEP (Figure 6e,g). In contrast, grazing and aboveground biomass did not directly affect ER and SR (Figure 6b,d), but they did directly and indirectly (via reductions in ammonium nitrogen) reduce belowground biomass, which influenced the ER and SR rate (Figure 6f,h).

## 4 DISCUSSION

### 4.1 Effect of grazing intensity on net ecosystem CO_2_ exchange

Grazing alters the balance between carbon sources and sinks in desert steppe (de la Motte et al., 2018; Ondier et al., 2021). Our finding that NEE, ER, and GEP significantly decreased under heavy grazing intensity is consistent with previous results from desert steppe (Jin et al., 2023; Wang et al., 2023), in our case, due to a reduction in the aboveground biomass of plants (Figure 6, Figure S4), grazing livestock reduces the aboveground biomass through foraging, which reduces the effective photosynthetic area. Previous studies have also indicated that grazing reduced CO_2_ exchange by reducing aboveground biomass (Danielewska et al., 2015; Ondier et al., 2021; Xu et al., 2022). Some studies have suggested that grazing appears to have little influence on the carbon budget of grassland ecosystems (Dai et al., 2014; Fang et al., 2010), although the NEE rate can be enhanced in the short‐term grazing due to the compensatory growth of plants, resulting in a negligible impact on the carbon balance. However, in this study, after a long period of grazing (16 years), livestock feeding and trampling can cause both aboveground and belowground biomass depletion (Zhang et al., 2018). This may be related to the legacy effects of grazing (Zhang, Bennett, et al., 2023; Zhang, Zheng, et al., 2023). Decreased aboveground biomass of heavily grazed plants due to long‐term grazing effects, the amount of leaf area available for both photosynthesis and respiration is reduced, leading to decreases in the net CO_2_ exchange rate (Oba et al., 2000; Shi et al., 2022). Interestingly, we found no significant difference in NEE rates between the light and moderate grazing treatments (Figure 4a), likely because these treatments did not influence aboveground biomass and plant cover (Figure 1a, Figure S5).

Furthermore, we also found a positive correlation between shrubs and semi‐shrubs biomass and NEE (Figures 5c and 6a, Figure S4a), which is consistent with previous studies (Zhao et al., 2021). The likely reason for this is that due to grazing sheep feeding preferences, a large number of shrubs and semi‐shrubs are being taken, and carbon substrate for photosynthesis is being consumed, which reduces the effective photosynthetic area and consequently inhibits carbon exchange (Oba et al., 2000). Sheep tend to prefer grazing on shrubs and semi‐shrubs, which are palatable and protein‐rich (Guo et al., 2021). Grasses, such as the dominant *Stipa breviflora* (Liu, Han, et al., 2015), are not preferred by livestock at our study site, and shrubs and semi‐shrubs were strongly influenced by grazing (Han & Biligetu, 2004; Li et al., 2011). Alternatively, shrub roots can reach up to 70 cm deep into the soil layer, allowing them to better utilize deeper water and nutrients (Tan et al., 2009), which can help maintain a high carbon fixation capacity and a high net carbon uptake capacity (Li et al., 2020; Niu et al., 2023). Indeed, in our study area, the photosynthetic efficiency of shrubs and semi‐shrubs is higher than that of other plant functional groups (Wang, 2023), which may explain why their loss dramatically reduces NEE.

The mechanisms of nitrogen uptake and utilization in plants are complex (Schimel et al., 2001). In this study, we found that the total N content of the plant community was significantly reduced in the MG treatment, but there was no difference in the HG treatment compared to the control, which is supported by previous studies (Hou et al., 2020; Song et al., 2018). This is likely because, in the HG treatment, livestock has a long‐term impact by trampling and foraging, which removes senescent branches and leaves while stimulating the redistribution of nitrogen to younger plant parts, ultimately resulting in no change in plant community N content (Liu et al., 2023; Wang et al., 2022). Further, our finding that plant N content is negatively correlated with net ecosystem CO_2_ exchange is inconsistent with previous findings that loss of leaf N attenuates ecosystem carbon cycling (Gong et al., 2021; Wang, Fu, et al., 2014). This may be due to changes in N partitioning that affect the ratio of N content in leaves between photosynthetic and nonphotosynthetic organs. While many studies have shown that plant nitrogen content is closely related to photosynthetic rate, the mechanism of its influence needs to be considered along with external disturbances such as grazing (Hikosaka, 2004). Long‐term grazing tends to induce shifts in plant ecological strategy toward more stress tolerators (Zheng et al., 2024). These plants allocate more nitrogen to nonphotosynthetic proteins. Although this increases the resistance of leaves to ensure their own compensatory growth, it reduces the photosynthetic capacity of plants (Onoda et al., 2004), causing a decrease in the rate of net CO_2_ exchange.

### 4.2 Effect of grazing intensity on soil respiration

As the second‐largest flux between terrestrial ecosystems and the atmosphere, soil respiration contributes 60%–90% of the total respiration of terrestrial ecosystems (Aanderud et al., 2011). Because desert steppe is sparsely vegetated, soil respiration is a particularly important determinant of carbon balance in this ecosystem. We found that grazing influenced soil respiration rates (Figure 4d), as has been found previously (Wang et al., 2020). Likewise, we found that belowground biomass was correlated with soil respiration (Figure 6, Figure S4), as has been shown elsewhere (Diao et al., 2022; Pregitzer et al., 2008; Wu et al., 2016). As a result, grazing leads to reduced respiration rates due to losses of both aboveground and belowground plant biomass, inhibition of plant root growth, and severe dissipation of soil organic matter (Cao et al., 2004; Mei et al., 2018). In addition, lower belowground biomass leads to fewer released secretions at the interroot level, which provides an unfavorable environment for soil microbial respiration, further inhibiting soil respiration (Li et al., 2013; Wu et al., 2016).

Nitrogen is the most important nutrient for plant growth (LeBauer & Treseder, 2008), and its addition can stimulate soil respiration in nutrient‐poor conditions (Smith, 2005). The low precipitation during our study period, coupled with heavy grazing, likely led to severe limitation of soil nitrogen, which slowed down competition between above‐ and belowground plants productivity and reduced soil respiration (Kuzyakov & Xu, 2013), which can otherwise increase soil respiration (Song et al., 2021). We found that soil ammonium nitrogen was positively correlated with soil respiration (Figure 6h, Figure S4b) and that the conversion of ammonium to available nitrogen can directly influence on plant productivity and ultimately, especially plant belowground productivity, soil respiration. This is because the affinity of dissolved oxygen and aeration tissue for ${\mathrm{NH}}_{4}^{+}$ and NO_3_^−^ in root respiration mainly depends on ${\mathrm{NH}}_{4}^{+}$ availability (Cao et al., 2020), enhanced glutamate dehydrogenase regulation after ${\mathrm{NH}}_{4}^{+}$ uptake, the enhancement of glutamate dehydrogenase regulation and amino acid metabolic reactions increases root N use efficiency and promotes root growth (Knapp et al., 2017). Thus, the change in soil ammonium Nitrogen content is a main factor influencing soil respiration (Gong et al., 2021; Onoda et al., 2004).

### 4.3 Effects of climate variables on ecosystem carbon exchange and soil respiration

Grazing by livestock influences the productivity and stability of grassland ecosystems, which in turn generates feedback mechanisms on the carbon cycle. However, external environmental factors can moderate this process (Liang et al., 2020), such as precipitation, which largely regulates ecosystem carbon exchange (Liang et al., 2017). In our study site, a 5‐year ecosystem carbon exchange experiment showed that grazing reduced NEE less in wetter than in drier years (Jin et al., 2023), such that precipitation causes divergent responses of whether long‐term grazing influences the carbon sink. As expected, we found that NEE, ER, and GEP were all influenced by precipitation levels (Figure S3). Moisture limits carbon exchange in desert grassland ecosystems since when water is lost from the plant, the plant closes its stomata and thus reduces transpiration and also reduces the diffusion of CO_2_ into the interior of the leaves, which ultimately affects photosynthetic carbon fixation (Jobbagy et al., 2002; Pan et al., 2008).

The positive correlations we found between soil moisture and SR and ER (Figures S2d and S4b) emerge because moisture influences surface productivity, root distribution, soil microbial activity, and nutrient availability. When soil moisture is more significant, this likely promotes the growth of plant roots to enhance microbial activity and promote organic matter decomposition (Helfter et al., 2015; Peng et al., 2015), as has been shown previously in desert steppe (Jin et al., 2023; Wang et al., 2023).

Likewise, variation in soil temperature influences ecosystem carbon exchange mainly by affecting GEP and ER (Chen et al., 2023; Ganjurjav et al., 2018; Li et al., 2019; Luo et al., 2001). However, consistent with our results showing a minimal influence of temperature on ecosystem carbon exchange in a desert steppe (Figure S3a), Wu et al. (2011) noted that elevated temperatures increase grass ER rates, we did find that variation in soil temperature contributed to ER, Wu et al. (2021) found similar results in a 12‐year study. Finding the optimum temperature and ER may contribute to homeostasis for ecosystem C balance between fluxes (Chen et al., 2023).

## 5 CONCLUSIONS

In this study, we assessed the impact of different levels of grazing intensity as well as the associated direct and indirect effect factors on ecosystem carbon exchange and soil respiration. Over the course of the growing season, we found that the desert steppe remained in a state of carbon uptake (carbon sink) following 16 years of continuous grazing. Our study shows that grazing decreased net ecosystem carbon exchange by decreasing aboveground biomass, especially the functional group of shrubs and semi‐shrubs biomass. At the same time, belowground biomass and soil ammonium nitrogen influenced soil respiration under grazing. Our results provide deeper insights for understanding the relationships between ecosystem CO_2_ exchange, plant biomass, and soil nutrients, which could inform research on the carbon sequestration potential of grassland.

## AUTHOR CONTRIBUTIONS

**Xin Ju:** Data curation (equal); formal analysis (lead); investigation (lead); methodology (lead); resources (equal); software (lead); visualization (lead); writing – original draft (lead); writing – review and editing (lead). **Bingying Wang:** Data curation (equal). **Lianhai Wu:** Supervision (lead). **Xiaojia Zhang:** Data curation (equal). **Qian Wu:** Conceptualization (lead); funding acquisition (lead); project administration (lead); supervision (lead); validation (lead); writing – review and editing (lead). **Guodong Han:** Conceptualization (lead); funding acquisition (lead); project administration (lead); supervision (lead); validation (lead).

## CONFLICT OF INTEREST STATEMENT

The authors declare no conflicts of interest.

## Supporting information


Appendix S1

Data S1

## Data Availability

The data that support the findings of this study are available in the Supporting Information of this article. The additional data that support the findings of this study are available from the corresponding author upon reasonable request. Additional supporting information can be found online in the Supporting Information section at the end of this article.
