# Supplementary material for: Grazing decreases net ecosystem carbon exchange by decreasing shrub and semi‐shrub biomass in a desert steppe
Source: Ecol Evol. 2024 Jun 25;14(6):e11528. doi: 10.1002/ece3.11528 (PMC11199334; doi:10.1002/ece3.11528)
Supplement: Supplementary file 1 — Appendix S1 [file ECE3-14-e11528-s002.docx]

**Supplementary Material**

**Table**

**Table S1.** Basic information on plant species and plant functional groups during 2020 at the study site.

| **Latin name of specie**s | **Plant functional groups** |
| --- | --- |
| *Stipa breviflora* Griseb. | perennial grass |
| *Stipa krylovii* Roshev. | perennial grass |
| *Cleistogenes songorica* (Roshev.) Ohwi. | perennial grass |
| *Leymus chinensis* (Trin.) Tzvel. | perennial grass |
| *Cleistogenes squarrosa* (Trin.) Keng. | perennial grass |
| *Agropyron cristatum*(L.) Gaertn. | perennial grass |
| *Convolvulus ammannii* Desr. | perennial forb |
| *Allium tenuissimum* L. | perennial forb |
| *Allium mongolicum* Regel. | perennial forb |
| *Astragalus galactites* Pall. | perennial forb |
| *Lagochilus ilicifolius* Bunge ex Benth. | perennial forb |
| *Carex pediformis* C. A. Mey*.* | perennial forb |
| *Aster altaicus*Willd. | perennial forb |
| *Cymbaria daurica* L. | perennial forb |
| *Allium ramosum*L. | perennial forb |
| *Iris tenuifolia* Pall. | perennial forb |
| *Sibbaldianthe bifurca* (L.) Kurtto & T. Erikss. | perennial forb |
| *Artemisia frigida* Willd. | subshrub |
| *Caragana microphylla* Lam. | shrub |
| *Caragana stenophylla* Pojark. | shrub |
| *Bassia prostrata*(L.) Beck. | subshrub |
| *Kali collinum* (Pall.) Akhani & Roalson. | annual and biennial plant |
| *Neopallasia pectinata* (Pall.) Poljak. | annual and biennial plant |
| *Artemisia scoparia* Waldst. et Kit. | annual and biennials plant |
| *Chenopodium glaucum* L. | annual and biennial plant |
| *Teloxys aristata* (L.) Moq. | annual and biennial plant |
| *Euphorbia humifusa*Willd. | annual and biennial plant |
| *Lappula myosotis*Moench. | annual and biennial plant |

**Figure**

**Figure S1.** Daily mean air temperature (lines) and daily precipitation (bars) in the growing seasons in 2020**.**

**Figure S2.** Soil temperature (a) and soil moisture (b) at 10 cm soil depth under different grazing intensity in growing seasons. The figure shows the differences in soil temperature and moisture under different grazing intensities, and the inset shows the average levels of soil temperature and soil moisture during the growing season in 2020 and the differences between treatments, different lowercase letters indicate significant differences between treatments (*p*<0.05). Codes of different treatments are as follows: CK, control / no grazing; LG, light grazing; MG, moderate grazing; HG, heavy grazing.

**Figure S3.** Correlations between air temperature (a), precipitation (b), soil temperature (c), soil moisture (d) and ecosystem CO_2_ fluxes (NEE, ER, GEP, SR) in the growing season of 2020.

**Figure S4.** Pearson’s correlation coefficient among NEE, ER, GEP, SR, and plant factors (a) and soil factors (b). The significance of correlation coefficients (positive or negative) is indicated by color intensity changing from red to blue. The circles show significant relationships, * *p*<=0.05, ** *p*<=0.01, *** *p*<=0.001. NEE: net ecosystem CO2 exchange, GEP: gross ecosystem productivity, ER: ecosystem respiration, SR: soil respiration, AGB: aboveground biomass; BGB: belowground biomass; PG: perennial grass biomass; SS: shrub and semi-shrub biomass; PF: perennial forb biomass; AB: annual and biennial plant biomass; PTC: plant total carbon; PTN: plant total nitrogen, TC, total carbon; TN, total nitrogen; TP, total phosphorus; SOC, organic carbon; AN, ammonium nitrogen; AP, available phosphorus; MBC, microbial biomass carbon; MBN, microbial biomass nitrogen; SM: soil moisture; ST: soil temperature.

**Figure S5.** Plant coverage under different grazing intensity in growing seasons. The figure shows the differences in plant coverage under different grazing intensities, and the inset shows the average levels of plant coverage during the growing season in 2020 and the differences between treatments, different lowercase letters indicate significant differences between treatments (*p*<0.05). Codes of different treatments are as follows: CK, control / no grazing; LG, light grazing; MG, moderate grazing; HG, heavy grazing.





**Figure S1.** Daily mean air temperature (lines) and daily precipitation (bars) in the growing seasons in 2020**.**





**Figure S2.** Soil temperature (a) and soil moisture (b) at 10 cm soil depth under different grazing intensity in growing seasons. The figure shows the differences in soil temperature and moisture under different grazing intensities, and the inset shows the average levels of soil temperature and soil moisture during the growing season in 2020 and the differences between treatments, different lowercase letters indicate significant differences between treatments (*p*<0.05). Codes of different treatments are as follows: CK, control / no grazing; LG, light grazing; MG, moderate grazing; HG, heavy grazing.





**Figure S3.** Correlations between air temperature (a), precipitation (b), soil temperature (c), soil moisture (d) and ecosystem CO_2_ fluxes (NEE, ER, GEP, SR) in the growing season of 2020.





**Figure S4.** Pearson’s correlation coefficient among NEE, ER, GEP, SR, and plant factors (a) and soil factors (b). The significance of correlation coefficients (positive or negative) is indicated by color intensity changing from red to blue. The circles show significant relationships, * *p*<=0.05, ** *p*<=0.01, *** *p*<=0.001. NEE: net ecosystem CO2 exchange, GEP: gross ecosystem productivity, ER: ecosystem respiration, SR: soil respiration, AGB: aboveground biomass; BGB: belowground biomass; PG: perennial grass biomass; SS: shrub and semi-shrub biomass; PF: perennial forb biomass; AB: annual and biennial plant biomass; PTC: plant total carbon; PTN: plant total nitrogen, TC, total carbon; TN, total nitrogen; TP, total phosphorus; SOC, organic carbon; AN, ammonium nitrogen; AP, available phosphorus; MBC, microbial biomass carbon; MBN, microbial biomass nitrogen; SM: soil moisture; ST: soil temperature.





**Figure S5.** Plant coverage under different grazing intensity in growing seasons. The figure shows the differences in plant coverage under different grazing intensities, and the inset shows the average levels of plant coverage during the growing season in 2020 and the differences between treatments, different lowercase letters indicate significant differences between treatments (*p*<0.05). Codes of different treatments are as follows: CK, control / no grazing; LG, light grazing; MG, moderate grazing; HG, heavy grazing.
